# Exploration of intermediate-sized INDELs by next-generation multigene panel testing in Han Chinese patients with breast cancer

**DOI:** 10.1038/s41439-019-0080-8

**Published:** 2019-10-29

**Authors:** Chihiro Hata, Hirofumi Nakaoka, Yu Xiang, Dong Wang, Anping Yang, Dahai Liu, Fang Liu, Qingfeng Zou, Ke Zheng, Ituro Inoue, Hua You

**Affiliations:** 1 Human Genetics Laboratory, National Institute of Genetics, Mishima, Japan; 2 The Department of Clinical Laboratory, The First Affiliated Hospital of Chongqing Medical University, Chongqing, China; 3 Chongqing University Cancer Hospital & Chongqing Cancer Institute & Chongqing Cancer Hospital, Chongqing, China; 4 School of Stomatology and Medicine, Foshan University, Foshan, Guangdong, China; 5 Affiliated Cancer Hospital & Institute of Guangzhou Medical University, Guangzhou, China; 6 Department of Endocrine and Breast Surgery, The First Affiliated Hospital of Chongqing Medical University, Chongqing, China; 7 Affiliated Hospital of Academy of Military Medical Sciences, Beijing, China

**Keywords:** Genetic testing, Genetics research

## Abstract

Multigene panel testing via next-generation sequencing focuses on the detection of small-sized mutations, such as single nucleotide variants and short insertions and deletions (INDELs). However, intermediate-sized INDELs have not been fully explored due to technical difficulties. Here, we performed bioinformatics analyses to identify intermediate-sized INDELs in 54 cancer-related genes from 583 Han Chinese patients with breast cancer. We detected a novel deletion–insertion in a translational variant of *PTEN* (also known as *PTENα*) in one patient.

Breast cancer is the most common type of cancer among women^1^, and approximately 10–15% of the cases are associated with hereditary mutations in DNA repair genes, including *BRCA1/2*^2^. With the advent of next-generation sequencing (NGS) technologies, genetic testing of *BRCA1/2* is now conducted worldwide. Multigene panel testing utilizing NGS technologies has enabled researchers to identify pathogenic mutations in genes other than *BRCA1/2*. It is also useful for identifying associations between germline mutations and clinicopathological characteristics. For example, it has been demonstrated that germline mutations in the genes involved in homologous recombination pathways, such as *BARD1*, *BRCA1*, *BRCA2*, *PALB2*, and *RAD51D*, are strongly associated with triple-negative breast cancer^3^.

Most applications of NGS-based multigene panel testing focus only on small-sized variants containing single nucleotide variants (SNVs) and short insertions and deletions (INDELs). In addition, high-risk patients with hereditary breast and ovarian cancers harbor large germline rearrangements in *BRCA1/2*^4^. The effects of intermediate-sized INDELs (50–10,000 bp) on the pathogenicity of breast cancer remain uninvestigated due to technical difficulties in analyzing NGS data^5,6^. However, intermediate-sized INDELs are possibly involved in the pathology of breast cancer. Therefore, to clarify the clinical significance of intermediate-sized INDELs in breast cancer, we attempted to identify intermediate-sized INDELs in 54 cancer predisposition genes among 583 Han Chinese patients with breast cancer and identified a novel deletion–insertion in a translational variant of *PTEN* (also known as *PTENα* or *PTEN-Long*) in one patient.

Information regarding the study subjects and target-gene sequencing has been described in our previous study (Hata et al., submitted). In brief, 583 Han Chinese patients with breast cancer were recruited between December 2016 and September 2017 at the First Affiliated Hospital of Chongqing Medical University and Affiliated Cancer Hospital and Institute of Guangzhou Medical University. All patients provided informed consent for participation in this study. The Ethics Committees of the First Affiliated Hospital of Chongqing Medical University, the Affiliated Cancer Hospital & Institute of Guangzhou Medical University, and the National Institute of Genetics approved the study protocols. The patients’ mean age at diagnosis was 49.1 (standard deviation: 9.2) years.

Fifty-four cancer predisposition genes were selected based on previous studies of multigene panel testing for hereditary breast and/or ovarian cancer (Table S1). Target sequencing of these genes was performed using the precapture pooling method described in previous studies by using DNA samples isolated from peripheral blood^7,8^. The libraries were sequenced on an Illumina HiSeq 2500 platform operating in rapid-run mode using a 2 × 100-bp paired-end protocol (Illumina, San Diego, CA, USA).

NGS data processing and variant calling were performed using BWA^9^ and GATK^10,11^. Functional annotation was implemented using ANNOVAR^12^. The estimation of variant frequencies in general populations was based on publicly available databases provided by ExAC^13^. Nonsense and splice-site SNVs and frameshifting INDELs were considered pathogenic. The variants with previously established pathogenic or benign effects were explored based on ClinVar^14^. We attempted to detect intermediate- to large-sized INDELs from mapped paired-end sequencing reads via bioinformatics analysis using Manta^15^.

The average depth for the target regions was 117.6, and the mean proportion of the targeted regions covered by at least 20 reads was 99.3%, supporting confident variant detection. We identified 78 pathogenic mutations (43 SNVs and 35 short INDELs) in 21 genes containing *BRCA1/2* (Hata et al., submitted). However, pathogenic SNVs and short INDELs were not detected in 85.8% (500/583) of the patients.

Using Manta, we identified two intermediate-sized INDELs from patients without pathogenic SNVs or short INDELs. One was an 89-bp heterozygous deletion present in intron 14 of *APC* (NM_000038.5:c.1743 + 15_1743 + 103del89). The patient with this mutation was 53 years old and diagnosed with triple-negative breast cancer because estrogen receptor (ER), progesterone receptor (PR), and human epidermal growth factor receptor type 2 (HER2) were negative in immunohistochemistry (IHC) and fluorescence in situ hybridization. Adenomatosis polyposis coli (APC) is a tumor suppressor protein that acts as an antagonist of the Wnt signaling pathway. A lack of this gene causes familial adenomatous polyposis (OMIM175100). The identified intermediate-sized deletion in the intronic region of *APC* might change the splicing behavior of the gene. However, this deletion is registered as “likely benign” in ClinVar. The allele frequency of this deletion is 0.9% in the general East Asian population from the ExAC project. Based on these findings, the significance of this deletion for the pathogenicity does not seem high.

The other INDEL was a combination of a 47-bp deletion and a 68-bp insertion in *PTEN* (Fig. 1a) [NM_001304717.5(PTEN_v001):c.8_54delinsAGTAATGTTAGCGGTTAGGCGTACGGCCAGGGCTATTGGTTGAATGAGTAGGCTGATGGTTTCGATAG (p. Arg3_Pro18delinsGlnTer); (Fig. 1a–d)]. Transcript NM_00130417 is a translational variant of *PTEN* (hereafter referred to as *PTENα*). The patient was heterozygous for this deletion–insertion. Although the result from Manta suggested the presence of deletion–insertion at *PTENα*, we could not exclude the possibility that the detected deletion–insertion was a false positive finding. Furthermore, the results from Manta did not provide any information about the possible source of the inserted sequence. To clarify these issues, we conducted additional bioinformatics analyses as described below. In addition, we performed Sanger sequencing to verify breakpoints of the deletion–insertion.

**Fig. 1:**
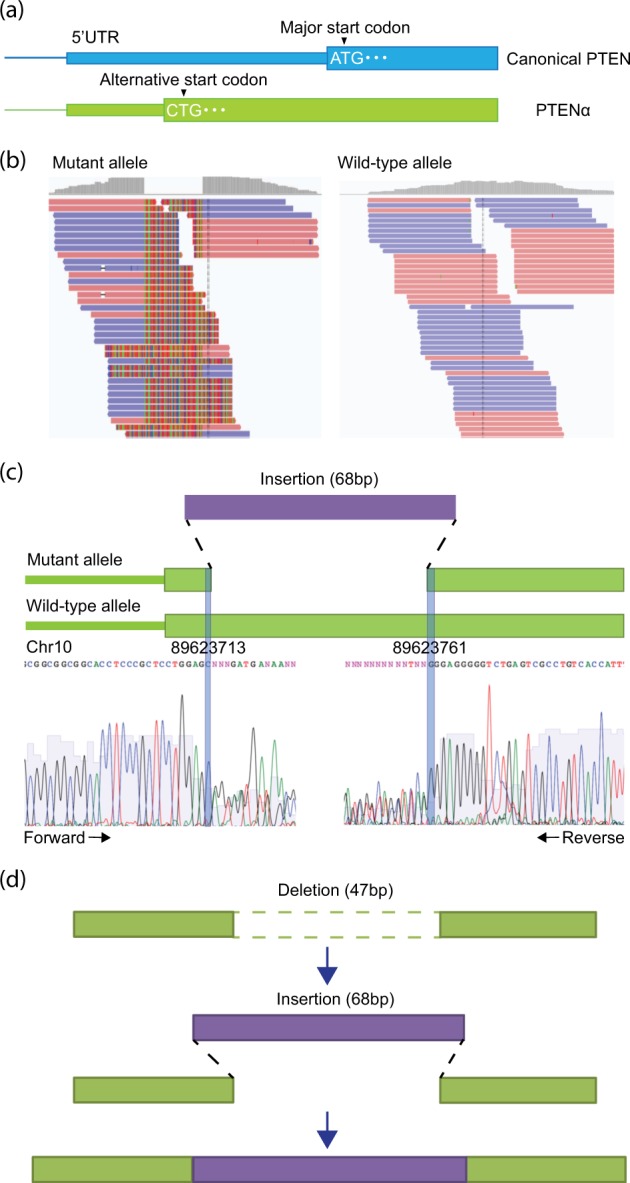
Overview of the novel intermediate-sized INDEL in *PTENα*. **a** Differences in the structure between canonical *PTEN* (top) and *PTENα* (bottom). *PTENα* has an alternative start codon (CTG). **b** Detection of deletion junctions in *PTENα*. The alignment result of soft-clipped reads derived from the mutant allele (i.e., deletion–insertion) is shown. For comparison, the alignment result of reads that were not soft-clipped from the wild-type allele is also shown. The sequences color-coded by light red and blue indicate the sequences matched with that of the human reference genome. Highlighted bases indicate the sequences that are mismatched with the reference genome (i.e., inserted sequences). **c** A schematic representation with plausible junctions of the deletion–insertion in *PTENα*. The purple bar indicates an inserted sequence (68 bp), whereas the light green bars indicate *PTENα* sequences. Sanger sequencing confirmed the breakpoints of the deletion–insertion. Sanger sequencing using forward (left) and reverse (right) primers revealed aberrant electropherograms after the breakpoints of the deletion–insertion because the fluorescent signals from mutant and wild-type alleles were mixed. The breakpoints of the deletion–insertion are depicted as blue vertical lines. Sanger sequencing was performed using the BigDye Terminator v3.1 Cycle Sequencing Kit (Thermo Fisher Scientific, Waltham, MA, USA) on the ABI 3130x Genetic Analyzer (Applied Biosystems, Foster City, CA, USA). The oligonucleotide sequences of the PCR primers are shown in Table S2. **d** A plausible mechanism of deletion and insertion at the same position. Light green and purple sequences indicate the reference and inserted sequences, respectively. Double-strand DNA breaks may result in a 47-bp deletion accompanied by a 68-bp insertion

We attempted to determine the junctions of the intermediate-sized INDELs via in-house bioinformatics analysis that leverages split-reads of paired-end sequencing. First, we extracted soft-clipped reads containing a part of the unmatched sequence with the reference genome. Second, we divided the soft-clipped reads into unmatched and matched sequences by using an in-house Perl script. Third, we aligned these two types of read sequences with the reference genome by using BWA^9^. Finally, we searched the genomic positions where these reads were mapped and successfully determined the deletion junctions at *PTENα* from the realignment of the matched sequences of the soft-clipped reads (Fig. 1b, c). The presence of the unmatched sequences of the soft-clipped reads at the deletion junctions indicated that a DNA fragment derived from another region was inserted into the *PTENα* deletion site (Fig. 1d).

Next, we investigated the origin of the inserted DNA fragment (Fig. 2) by assembling the unmatched sequences of the soft-clipped reads at the deletion site of *PTENα* to determine a plausible sequence of the inserted DNA fragment. We then searched for the sequence against the human genome by using BLAT^16^. As a result, the inserted sequence matched two candidate regions: (i) a reverse complement of a region [chr1:569503–569570 (hg19)] within the nuclear mitochondrial sequence (chr1:564465–570304)^17^ and (ii) a reverse complement of a part of the mitochondrial genome (chrM:8955–9022). Because the combinations of either of the two candidate-inserted segments with the PTENα sequence based on the human reference genome could not accurately account for the observed deletion–insertion, it resulted in one unresolved mismatch (G allele). Based on these results, we developed two hypotheses about the structure of the deletion–insertion. Hypothesis 1 assumed a 46-bp deletion and 67-bp insertion, in which the G allele originated from an alteration at the breakpoint of *PTENα*, whereas hypothesis 2 assumed a 47-bp deletion and 68-bp insertion, in which the G allele originated from an alteration in either of the two candidate insertions. Therefore, we scrutinized common genetic polymorphisms deposited in dbSNP. As a consequence, hypothesis 1 was rejected because there was no genetic polymorphism rendering the G allele at the breakpoint of *PTENα* (Fig. 2b). When considering hypothesis 2, the two candidate insertions were identical; however, a SNP located in the nuclear mitochondrial sequence on chromosome 1 (rs1198320487: NC_000001.10:g.569503T>C) differentiated the sequences (Fig. 2c). Finally, we determined that the G allele originated from the alternative allele of rs1198320487 in the nuclear mitochondrial sequence on chromosome 1.

**Fig. 2:**
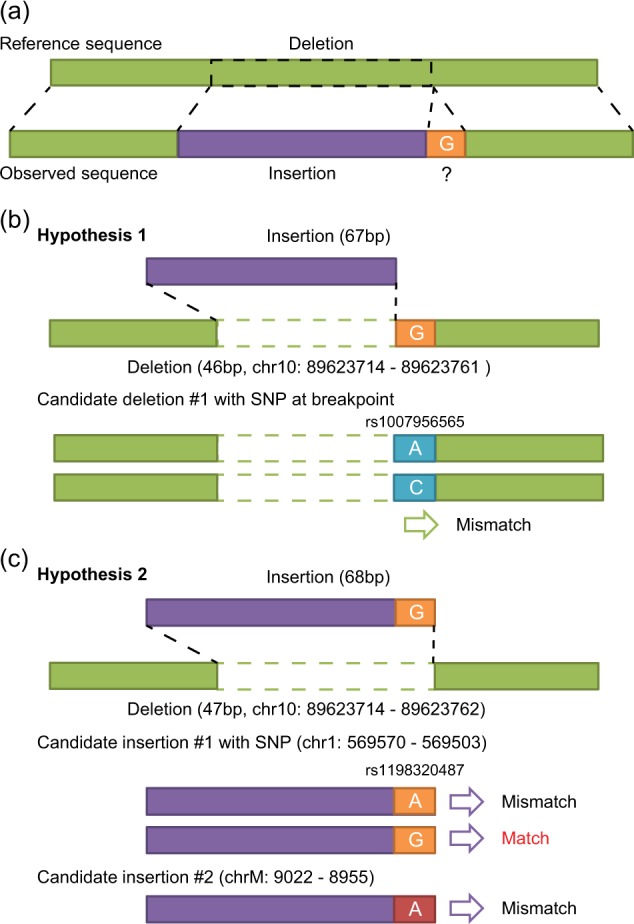
Inference on the origin of the inserted sequence by the two hypotheses. **a** Differences in the structure between the reference (top) and observed (bottom) sequences. Light green and purple sequences indicate the reference and inserted sequences, respectively. The unresolved mismatch (G allele) around the breakpoint of *PTENα* is highlighted by an orange box. **b** Hypothesis 1 for the origin of the unresolved G allele. Hypothesis 1 assumes a 46-bp deletion and 67-bp insertion, in which the G allele arises from an alteration in the *PTENα* sequence by an SNP (rs1007956565, A/C). There are two possible sequences by the SNP (rs1007956565) at the breakpoint of *PTENα*; however, these two sequences cannot account for the unresolved mismatch (G allele). **c** Hypothesis 2 for the origin of the unresolved G allele. Hypothesis 2 assumes a 47-bp deletion and 68-bp insertion, in which the G allele arises from an alteration within the inserted sequence (purple). The two candidate sources of the inserted sequence are as follows: (i) a reverse complement of a region (chr1:569503–569570) within a nuclear mitochondrial sequence (chr1:564465–570304) and (ii) a reverse complement of a part of the mitochondrial genome (chrM: 8955–9022). These two candidate regions have identical sequences; however, there is a SNP (rs1198320487, A/G on the reverse strand) in the nuclear mitochondrial sequence on chromosome 1. One of the two possible sequences by the SNP (rs1198320487) can account for the unresolved mismatch (G allele). As a result, the source of the inserted sequence is likely to be the reverse complement of a region (chr1:569503–569570) within the nuclear mitochondrial sequence (chr1:564465–570304) with the alternative G allele at the SNP rs1198320487 site

The patient with the deletion–insertion in *PTENα* was diagnosed at 42 years of age, which is earlier than the average age of diagnosis in this study. IHC of ER and PR were negative, and we could not retrieve the results of HER2 from the patient’s clinical charts. The frequency of the deletion–insertion of *PTENα* was not observed in any of the ExAC and other publicly available populations. The deletion–insertion was also not registered in either dbSNP or ClinVar, indicating that this mutation was a novel germline mutation. The identified deletion–insertion on exon 1 of *PTENα* was predicted to create a stop codon at the fourth amino acid of the PTENα protein.

*PTEN* is a tumor suppressor gene. *PTEN* mutations are commonly found in patients with inherited cancer syndromes, such as Cowden syndrome (OMIM158350). PTENα is a translational variant of *PTEN* and has an additional 173 amino acids at the N-terminus, labeled as alternatively translated region (ATR) (Fig. 1a). PTENα prevents cancer growth by antagonizing phosphoinositide-3 kinase signaling as well as canonical *PTEN*. More importantly, ATR contains a protein-binding domain and a cleavage site. These regions allow *PTEN*α to bind to the cell membrane and be released into the extracellular space. Because ATR contains sequences that have homology with known cell-permeable peptides, *PTEN*α enters into and acts in neighboring cells. Furthermore, PTENα without the cleavage site could not suppress tumor cell growth compared with normal PTENα in vivo^18^. From these results, we assumed that this novel protein-truncating mutation in *PTENα* could lead to the development of breast cancer due to the lack of a tumor-suppressive function attributed to the PTENα protein. However, the functional significance of this deletion–insertion on canonical *PTEN* was unknown because the deletion–insertion was located on the 5′ untranslated region of canonical *PTEN* (Fig. 1a). Two pathogenic germline mutations on the N-terminal residues of *PTENα* were identified in a Chinese cohort of patients with autism spectrum disorder, although a definitive association between *PTENα* and neurodevelopment remains unknown^19^.

Based on telephonic interviews and clinical charts, the patient with the deletion–insertion in *PTENα* did not report any family history of breast or ovarian cancer. Although we could not obtain DNA samples from her family members, there is a possibility that the deletion–insertion was inherited through her father’s lineage. Other plausible explanations are that the mutation occurred de novo or arose at a very early stage of her development. Further examination of the genotypes of the mutation among her family members together with a review of her status is needed to assess the clinical significance of this novel deletion–insertion.

In conclusion, we identified a novel intermediate-sized deletion–insertion in *PTENα*, which can be a disease risk factor for breast cancer. This deletion–insertion may not be detected by general pipelines targeting SNVs and short INDELs in multigene panel testing. The breakpoint of the deletion and the possible source of the inserted fragment were determined by in-depth analyses. Therefore, our results suggest that patient-specific risk factors can be detected via detailed bioinformatics analyses.

## Supplementary information

Table S1

Table S2

## Data Availability

The relevant data from this Data Report are hosted at the Human Genome Variation Database at 10.6084/m9.figshare.hgv.2786, 10.6084/m9.figshare.hgv.2789.
