# Supplementary material for: Exploration of intermediate-sized INDELs by next-generation multigene panel testing in Han Chinese patients with breast cancer
Source: Hum Genome Var. 2019 Oct 29;6:51. doi: 10.1038/s41439-019-0080-8 (PMC6820797; doi:10.1038/s41439-019-0080-8)
Supplement: Supplementary file 1 — Table S1 [file 41439_2019_80_MOESM1_ESM.docx]

Table S1. The 54 breast cancer predisposition genes analyzed in this study.

| Gene name | Gene description | Associated diseases |
| --- | --- | --- |
| *BRCA1* | BREAST CANCER 1 GENE | Fanconi anemia; Breast-ovarian cancer; Pancreatic cancer |
| *BRCA2* | BRCA2 GENE | Fanconi anemia; Wilms tumor; Breast cancer; Breast-ovarian cancer; Glioblastoma; Medulloblastoma; Pancreatic cancer; Prostate cancer |
| *EPCAM* | EPITHELIAL CELLULAR ADHESION MOLECULE | Colorectal cancer; Diarrhea |
| *MLH1* | MutL, E. COLI, HOMOLOG OF, 1 | Colorectal cancer; Mismatch repair cancer syndrome; Muir-Torre syndrome |
| *MSH2* | MutS, E. COLI, HOMOLOG OF, 2 | Colorectal cancer; Mismatch repair cancer syndrome; Muir-Torre syndrome |
| *MSH6* | MutS, E. COLI, HOMOLOG OF, 6 | Colorectal cancer; Endometrial cancer; Mismatch repair cancer syndrome |
| *PMS1* | POSTMEIOTIC SEGREGATION INCREASED, S. CEREVISIAE, 1 | Breast cancer |
| *PMS2* | POSTMEIOTIC SEGREGATION INCREASED, S. CEREVISIAE, 2 | Colorectal cancer; Mismatch repair cancer syndrome |
| *AKT1* | V-AKT MURINE THYMOMA VIRAL ONCOGENE HOMOLOG 1 | Cowden syndrome |
| *ATM* | ATAXIA-TELANGIECTASIA MUTATED GENE | Ataxia-telangiectasia; Breast cancer |
| *BARD1* | BRCA1-ASSOCIATED RING DOMAIN 1 | Breast cancer |
| *BLM (RECQL3)* | BLM GENE (RECQ PROTEIN-LIKE 3) | Bloom syndrome |
| *BRIP1* | BRCA1-INTERACTING PROTEIN 1 | Breast cancer; Fanconi anemia |
| *CHEK2* | CHECKPOINT KINASE 2, S. POMBE, HOMOLOG OF | Li-Fraumeni syndrome; Breast and colorectal cancer, susceptibility to; Breast cancer, susceptibility to; Prostate cancer, familial, susceptibility to |
| *FANCC* | FANCC GENE | Fanconi anemia |
| *FANCM* | FANCM GENE | Premature ovarian failure; Spermatogenic failure |
| *GEN1* | GEN1, DROSOPHILA, HOMOLOG OF | Breast cancer |
| *MEN1* | MULTIPLE ENDOCRINE NEOPLASIA, TYPE I | Multiple endocrine neoplasia |
| *MRE11A* | MEIOTIC RECOMBINATION 11, S. CEREVISIAE, HOMOLOG OF, A | Ataxia-telangiectasia-like disorder 1 |
| *NBN* | NIBRIN | Aplastic anemia; Leukemia; Nijmegen breakage syndrome |
| *PALB2* | PARTNER AND LOCALIZER OF BRCA2 | Fanconi anemia; Breast cancer; Pancreatic cancer |
| *RAD50* | RAD50, S. CEREVISIAE, HOMOLOG OF | Nijmegen breakage syndrome-like disorder |
| *RAD51* | RAD51, S. CEREVISIAE, HOMOLOG OF | Fanconi anemia; Mirror movements; Breast cancer |
| *RAD51B* | RAD51, S. CEREVISIAE, HOMOLOG OF, B | Breast cancer |
| *RAD51C* | RAD51, S. CEREVISIAE, HOMOLOG OF, C | Fanconi anemia; Breast-ovarian cancer |
| *RAD51D* | RAD51, S. CEREVISIAE, HOMOLOG OF, D | Breast-ovarian cancer |
| *RECQL* | RECQ PROTEIN-LIKE | Breast cancer |
| *SLX4* | SLX4, S. CEREVISIAE, HOMOLOG OF | Fanconi anemia |
| *XRCC2* | X-RAY REPAIR, COMPLEMENTING DEFECTIVE, IN CHINESE HAMSTER, 2 | Fanconi anemia |
| *XRCC3* | X-RAY REPAIR, COMPLEMENTING DEFECTIVE, IN CHINESE HAMSTER, 3 | Breast cancer; Melanoma |
| *APC* | APC GENE | Adenomatous polyposis coli; Brain tumor-polyposis syndrome; Desmoid disease; Gardner syndrome |
| *ATR* | ATR GENE | Cutaneous telangiectasia and cancer syndrome; Seckel syndrome |
| *AXIN1* | AXIS INHIBITOR 1 | Caudal duplication anomaly |
| *AXIN2* | AXIS INHIBITOR 2 | Oligodontia-colorectal cancer syndrome |
| *BAP1* | BRCA1-ASSOCIATED PROTEIN 1 | Tumor predisposition syndrome |
| *BMPR1A* | BONE MORPHOGENETIC PROTEIN RECEPTOR, TYPE IA | Juvenile polyposis syndrome; Polyposis syndrome; Polyposis |
| *CDH1* | CADHERIN 1 | Blepharocheilodontic syndrome; Gastric cancer; Breast cancer; Prostate cancer |
| *CDK4* | CYCLIN-DEPENDENT KINASE 4 | Melanoma |
| *CDKN2A* | CYCLIN-DEPENDENT KINASE INHIBITOR 2A | Melanoma and neural system tumor syndrome; Orolaryngeal cancer; Pancreatic cancer/melanoma syndrome; Melanoma |
| *CTNNB1* | CATENIN, BETA-1 | Exudative vitreoretinopathy; Mental retardation |
| *FAM175A* | FAMILY WITH SEQUENCE SIMILARITY 175, MEMBER A | Breast cancer |
| *HOXB13* | HOMEOBOX B13 | Breast cancer |
| *MET* | MET PROTOONCOGENE | Deafness; Osteofibrous dysplasia |
| *MUTYH* | MutY, E. COLI, HOMOLOG OF | Adenomas; Colorectal adenomatous polyposis |
| *NF1* | NEUROFIBROMATOSIS, TYPE I | Neurofibromatosis |
| *PALLD* | PALLADIN, MOUSE, HOMOLOG OF | Pancreatic cancer |
| *PIK3CA* | PHOSPHATIDYLINOSITOL 3-KINASE, CATALYTIC, ALPHA | Cowden syndrome |
| *PPM1D* | PROTEIN PHOSPHATASE, MAGNESIUM/MANGANESE-DEPENDENT, 1D | Intellectual developmental disorder with gastrointestinal difficulties and high pain threshold |
| *PTCH1* | PATCHED, DROSOPHILA, HOMOLOG OF, 1 | Basal cell nevus syndrome; Holoprosencephaly |
| *PTEN* | PHOSPHATASE AND TENSIN HOMOLOG | Bannayan-Riley-Ruvalcaba syndrome; Cowden syndrome; Lhermitte-Duclos syndrome; Macrocephaly/autism syndrome; PTEN hamartoma tumor syndrome; VATER association with macrocephaly and ventriculomegaly; Glioma; Meningioma |
| *RET* | REARRANGED DURING TRANSFECTION PROTOONCOGENE | Central hypoventilation syndrome; Medullary thyroid carcinoma; Multiple endocrine neoplasia; Pheochromocytoma; Hirschsprung disease |
| *SMAD4* | MOTHERS AGAINST DECAPENTAPLEGIC, DROSOPHILA, HOMOLOG OF, 4 | Juvenile polyposis/hereditary hemorrhagic telangiectasia syndrome; Myhre syndrome; Polyposis |
| *STK11* | SERINE/THREONINE PROTEIN KINASE 11 | Pancreatic cancer; Peutz-Jeghers syndrome |
| *TP53* | TUMOR PROTEIN p53 | Adrenal cortical carcinoma; Breast cancer; Choroid plexus papilloma; Colorectal cancer; Hepatocellular carcinoma; Li-Fraumeni syndrome; Nasopharyngeal carcinoma; Osteosarcoma; Pancreatic cancer; Basal cell carcinoma; Glioma |

[1] Walsh T, Lee MK, Casadei S, Thornton AM, Stray SM, Pennil C et al. Detection of inherited mutations for breast and ovarian cancer using genomic capture and massively parallel sequencing. Proc Natl Acad Sci U S A. 2010; 107(28): 12629–12633. doi: 10.1073/pnas.1007983107

[2] Castéra L, Krieger S, Rousselin A, Legros A, Baumann JJ, Bruet O et al. Next-generation sequencing for the diagnosis of hereditary breast and ovarian cancer using genomic capture targeting multiple candidate genes. Eur J Hum Genet. 2014;22(11):1305-13. doi: 10.1038/ejhg.2014.16.

[3] Chong HK, Wang T, Lu HM, Seidler S, Lu H, Keiles S et al. The Validation and Clinical Implementation of BRCAplus: A Comprehensive High-Risk Breast Cancer Diagnostic Assay. PLoS One. 2014; 9(5): e97408. doi: 10.1371/journal.pone.0097408

[4] LaDuca H, Stuenkel AJ, Dolinsky JS, Keiles S, Tandy S, Pesaran T et al. Utilization of multigene panels in hereditary cancer predisposition testing: analysis of more than 2,000 patients. Genet Med. 2014; 16(11): 830–837. doi: 10.1038/gim.2014.40

[5] Couch FJ, Hart SN, Sharma P, Toland AE, Wang X, Miron P et al. Inherited Mutations in 17 Breast Cancer Susceptibility Genes Among a Large Triple-Negative Breast Cancer Cohort Unselected for Family History of Breast Cancer. J Clin Oncol. 2015; 33(4): 304–311. doi: 10.1200/JCO.2014.57.1414.

[6] Cybulski C, Carrot-Zhang J, Kluźniak W, Rivera B, Kashyap A, Wokołorczyk D et al. Germline RECQL mutations are associated with breast cancer susceptibility. Nat Genet. 2015;47(6):643-6. doi: 10.1038/ng.3284.

[7] Desmond A, Kurian AW, Gabree M, Mills MA, Anderson MJ, Kobayashi Y et al. Clinical Actionability of Multigene Panel Testing for Hereditary Breast and Ovarian Cancer Risk Assessment. JAMA Oncol. 2015;1(7):943-51. doi: 10.1001/jamaoncol.2015.2690.

[8] Easton DF, Pharoah PD, Antoniou AC, Tischkowitz M, Tavtigian SV, Nathanson KL et al. Gene-panel sequencing and the prediction of breast-cancer risk. N Engl J Med. 2015;372(23):2243-57. doi: 10.1056/NEJMsr1501341.

[9] Judkins T, Leclair B, Bowles K, Gutin N, Trost J, McCulloch J et al. Development and analytical validation of a 25-gene next generation sequencing panel that includes the BRCA1 and BRCA2 genes to assess hereditary cancer risk. BMC Cancer. 2015;15:215. doi: 10.1186/s12885-015-1224-y.

[10] Lincoln SE, Kobayashi Y, Anderson MJ, Yang S, Desmond AJ, Mills MA et al. A Systematic Comparison of Traditional and Multigene Panel Testing for Hereditary Breast and Ovarian Cancer Genes in More Than 1000 Patients. J Mol Diagn. 2015;17(5):533-44. doi: 10.1016/j.jmoldx.2015.04.009.

[11] Maxwell KN, Wubbenhorst B, D'Andrea K, Garman B, Long JM, Powers J et al. Prevalence of mutations in a panel of breast cancer susceptibility genes in BRCA1/2-negative patients with early-onset breast cancer. Genet Med. 2015;17(8):630-8. doi: 10.1038/gim.2014.176.

[12] Minion LE, Dolinsky JS, Chase DM, Dunlop CL, Chao EC, Monk BJ. Hereditary predisposition to ovarian cancer, looking beyond BRCA1/BRCA2. Gynecol Oncol. 2015;137(1):86-92. doi: 10.1016/j.ygyno.2015.01.537.

[13] Tung N, Battelli C, Allen B, Kaldate R, Bhatnagar S, Bowles K et al. Frequency of mutations in individuals with breast cancer referred for BRCA1 and BRCA2 testing using next-generation sequencing with a 25-gene panel. Cancer. 2015;121(1):25-33. doi: 10.1002/cncr.29010.

[14] Norquist BM, Harrell MI, Brady MF, Walsh T, Lee MK, Gulsuner S et al. Inherited Mutations in Women With Ovarian Carcinoma. JAMA Oncol. 2016;2(4):482-90. doi: 10.1001/jamaoncol.2015.5495.

[15] Tung N, Lin NU, Kidd J, Allen BA, Singh N, Wenstrup RJ et al. Frequency of Germline Mutations in 25 Cancer Susceptibility Genes in a Sequential Series of Patients With Breast Cancer. J Clin Oncol. 2016;34(13):1460-8. doi: 10.1200/JCO.2015.65.0747.
