# Supplementary material for: Exploration of intermediate-sized INDELs by next-generation multigene panel testing in Han Chinese patients with breast cancer
Source: Hum Genome Var. 2019 Oct 29;6:51. doi: 10.1038/s41439-019-0080-8 (PMC6820797; doi:10.1038/s41439-019-0080-8)
Supplement: Supplementary file 2 — Table S2 [file 41439_2019_80_MOESM2_ESM.docx]

Table S2. Primers for Sanger sequencing of the detected genes

| Target | Forward sequence (5' to 3') | Reverse sequence (5' to 3') |
| --- | --- | --- |
| PTENα | ATGTGGCGGGACTCTTTATG | GGAATGGGGAGAAGACGAAT |
| APC | TTTGCAGGTTATTGCGAGTG | TGCGGTACTCTAAAACTATGGACT |
